# Methylomic Analysis Identifies Frequent DNA Methylation of *Zinc Finger Protein 582 (ZNF582)* in Cervical Neoplasms

**DOI:** 10.1371/journal.pone.0041060

**Published:** 2012-07-16

**Authors:** Rui-Lan Huang, Cheng-Chang Chang, Po-Hsuan Su, Yu-Chih Chen, Yu-Ping Liao, Hui-Chen Wang, Yi-Te Yo, Tai-Kuang Chao, Hsuan-Cheng Huang, Ching-Yu Lin, Tang-Yuan Chu, Hung-Cheng Lai

**Affiliations:** 1 Department of Obstetrics and Gynecology, Tri-Service General Hospital, Taipei, Taiwan; 2 Institute of Biomedical Informatics, National Yang-Ming University, Taipei, Taiwan; 3 Laboratory of Epigenetics and Cancer Stem Cells, National Defense Medical Centre, Taipei, Taiwan; 4 Graduate Institute of Medical Sciences, National Defense Medical Center, Taipei, Taiwan; 5 Graduate Institute of Life Sciences, National Defense Medical Center, Taipei, Taiwan; 6 Department of Pathology, Tri-Service General Hospital, Taipei, Taiwan; 7 School of Medical Laboratory Science and Biotechnology, College of Medical Science and Technology, Taipei Medical University, Taipei, Taiwan; 8 Center for Cervical Cancer Prevention, Department of Obstetrics and Gynecology, Graduate Institute of Clinical Medicine, Tzu Chi Medical Center, Tzu Chi University, Hualien, Taiwan; Vanderbilt University Medical Center, United States of America

## Abstract

**Background:**

Despite of the trend that the application of DNA methylation as a biomarker for cancer detection is promising, clinically applicable genes are few. Therefore, we looked for novel hypermethylated genes for cervical cancer screening.

**Methods and Findings:**

At the discovery phase, we analyzed the methylation profiles of human cervical carcinomas and normal cervixes by methylated DNA immunoprecipitation coupled to promoter tiling arrays (MeDIP-on-chip). Methylation-specific PCR (MSP), quantitative MSP and bisulfite sequencing were used to verify the methylation status in cancer tissues and cervical scrapings from patients with different severities. Immunohistochemical staining of a cervical tissue microarray was used to confirm protein expression. We narrowed to three candidate genes: DBC1, PDE8B, and ZNF582; their methylation frequencies in tumors were 93%, 29%, and 100%, respectively. At the pre-validation phase, the methylation frequency of DBC1 and ZNF582 in cervical scraping correlated significantly with disease severity in an independent cohort (n = 330, both *P*<0.001). For the detection of cervical intraepithelial neoplasia 3 (CIN3) and worse, the area under the receiver operating characteristic curve (AUC) of ZNF582 was 0.82 (95% confidence interval  = 0.76–0.87).

**Conclusions:**

Our study shows ZNF582 is frequently methylated in CIN3 and worse lesions, and it is demonstrated as a potential biomarker for the molecular screening of cervical cancer.

## Introduction

Cervical cancer is one of the major causes of death in women with about 454,000 new cases and 200,000 deaths in 2010 worldwide [1]. Human papillomavirus (HPV) infection is the most important risk factor for cervical cancer [2]. However, although HPV infection is common in sexually active women, less than 1% of women infected with HPV progress to cervical cancer [3]. Other environmental, genetic, and/or epigenetic factors also play decisive roles in cervical carcinogenesis [4]–[8]. Papanicolaou (Pap) smears have been used for decades to screen for cervical cancer. The identification of cervical cancer and its precursor depends on the microscopic inspection of exfoliated cervical cells. However, cytological screening is associated with many problems, including its low sensitivity and high levels of bias [9]. Moreover, most countries in the world do not have the infrastructure for Pap screening. HPV infections are common. Although the detection of HPV as a surrogate marker of cervical cancer is sensitive, its specificity is low and the high numbers of false positive results entail unnecessary medical and psychological burdens [10], [11]. An alternative to HPV DNA testing are more specific novel biomarkers as p16(INK4a), ProEx C or HPV E6/E7 mRNA measuring the interaction of HPV with human cells [12]–[18]. Therefore, we need new markers for a better cervical cancer screening.

DNA methylation is one of the epigenetic mechanisms that influence gene transcription, chromatin structure, genomic stability, and the inactivation of imprinted genes and X chromosome [19]. 5′-Methylcytosine is prone to occur in the context of CG dinucleotides and is associated with transcriptional silencing when it occurs at promoter regions. Abnormal methylation of the promoters of tumor suppressor genes is common in various cancers, and the use of DNA methylation as a biomarker in clinical oncology is promising [20], [21].

Using a candidate gene approach identifies the association of DNA methylation in cervical cancer, and the analysis of genome-wide methylation has been rarely used to discover novel sites [7], [22]–[24]. The treatment of cervical cancer cell lines with demethylating agents, coupled to expression microarrays, has identified the genes encoding *SPARC* and *TFPI2* as highly methylated in invasive cervical cancer [25]. An approach based on restriction landmark genomic scanning (RLGS) identified the genes encoding *NOL4* and *LHFPL4* as methylated in cervical cancer [23]. The differential methylation hybridization (DMH) using a pilot methylation array identified *SOX1*, *NKX6-1*, *PAX1*, *WT1*, and *LMX1A* as frequently methylated genes in cervical cancer and its precursor lesions [22]. Further quantitative analysis of these genes demonstrated the possibility of using them to detect CIN3 and worse lesions from cervical scrapings [26]. With advances in epigenomic technology, more genes that are hypermethylated in cervical cancer may be detected. In this study, we compared normal cervical epithelium and cancer tissues, using methyl–DNA immunoprecipitation coupled to a high-density promoter tiling array (MeDIP-on-chip), to identify more genes hypermethylated in cancer as a discovery phase. We tested the clinical performance of these genes as biomarkers in a large independent cohort of cervical scrapings from patients with differing severities of the disease as a pre-validation phase.

## Methods

### Clinical samples

Between 1994 and 2008, we collected 57 cervical tumor tissues and 19 normal cervical cell scrapings for methylomic array analysis. The detail patient demographic is listed in Table S1. The quality of genomic DNA was analyzed with the Bioanalyzer 2100 (Agilent, CA, USA). Equal amounts of DNA from patients with the same histological diagnosis were pooled for immunoprecipitation. For a quick verification of the array data, twelve DNA pools (each pool contains five patients of the same histological diagnosis) were generated. Verified genes were further tested in a small-scale of clinical samples individually. We then enrolled 330 women for a cross-sectional pre-validation including patients' scraping cells, whose diagnosis is normal uterine cervix (*N = *156), cervical intraepithelial neoplasia 1 (CIN1) (*N* = 55), CIN2 (*N* = 31), CIN3/carcinoma *in situ* (CIS, *N* = 47), squamous cell carcinoma (SCC, *N* = 41). All patients were enrolled, diagnosed, treated, and their tissues banked at the National Defense Medical Center, Taipei, Taiwan, as described previously [24], [27]. The procurement, preservation and utilization of tissues in this study was approved and under the supervision of the institutional review board of the Tri-Service General Hospital. Informed consent was written and given by each patient providing specimens for collection.

### Cell lines

HeLa, SiHa, and CaSki cells cultured with or without demethylating agents were harvested for RNA and DNA isolation. HeLa cells were cultured in Dulbecco's modified Eagle's medium, and SiHa and CaSki cells were cultured in RPMI 1640. The media were supplemented with 10% (w/v) fetal bovine serum, 100 U/mL penicillin, 100 µg/mL streptomycin, and 2 mM l-glutamine (all media were from Invitrogen, Carlsbad, USA), and the cells were grown at 37°C in an atmosphere of 5% (v/v) CO_2_ in air. The cells were seeded at a density of 1×10^6^ cells per 100 mm dish and allowed to attach for 24 hours. They were then incubated in 5 µM 5-aza-2′-deoxycytidine (5-aza-dC; Sigma-Aldrich, Milwaukee, USA) for 4 days, with fresh 5-aza-dC added every day. On the fifth day, the cells were incubated in 0.3 µM trichostatin A (Sigma-Aldrich, Milwaukee, USA) for 24 hours.

### DNA extraction, RNA extraction, and bisulfite conversion

Genomic DNA was extracted from the cultured cells, scraped cells, or tumor tissues with the QIAmp DNA Mini Kit (QIAGEN GmbH, Hilden, Germany). We isolated the total RNA from cultured cells with the RNeasy Mini Kit (QIAGEN GmbH, Hilden, Germany). The genomic DNA (1 µg) was bisulfite modified using the CpGenome Fast DNA Modification Kit (Chemicon-Millipore, MA, USA), according to the manufacturer's recommendations, and dissolved in 70 µL of nuclease-free water.

### MeDIP-on-chip and Analysis

The detail about MeDIP-on-chip was listed in Material and Methods S1. Genomic DNA (10 µg) in 90 µL of nuclease-free water was fragmented by sonication to sizes of about 300–500 base pairs (bp). Anti-5-methylcytosine antibody (Abcam, ab1884, MA, USA) used to conjugate with Protein G Sepharose beads (Amersham GE, PA, USA) to pull down methylated DNA fragments and then detected on the HG18 CpG Promoter array (Roche NimbleGen, Penzberg, Germany). We used the HG18 CpG Promoter array (Roche NimbleGen, Penzberg, Germany) for hybridization. The labeling, array hybridization, scanning, and analysis were performed with the recommended NimbleGen equipment, according to the user guide version 1.0.

The signal intensity of each probe had a corresponding scaled log2 ratio. The log2 ratio was computed and scaled to center the ratio data around zero. Centering was performed by subtracting the biweight mean from the log2 ratio values for all probes. The peak data of the enrichments were analyzed according to the parameters: sliding window width  = 500 bp, *P*-value minimum cutoff (–log10) ≥2.0, and the default settings of NimbleScan software version 2.3 (Roche NimbleGen, Penzberg, Germany). Using the one-sided Kolmogorov–Smirnov (KS) test, *P-*values were analyzed to determine whether the probes were drawn from a significantly more positive distribution of intensity log2 ratios than those in the rest of the array. The resulting score of each probe was the –log10 value of *P-*value from the windowed KS test around that probe. We filtered the enrichments from the squamous cervical carcinoma (SCC), adenocarcinoma (AC), and normal samples separately, and then focused on those signals enriched in both SCC and AC, but not in the normal samples (Table S2). The enrichment of MeDIP signals was performed by KS test (–log10 *P-*value) and visualized with SignalMap version 1.9 (Figure S1). All raw microarray data were deposited in NCBI's Gene Expression Omnibus (GEO) under accession ID: GSE33187.

### Methylation-specific PCR (MSP) and bisulfite sequencing

The MSP primers and their optimal annealing temperatures are listed in Table S3. The condition for PCR reaction is shown in Material and Methods S1.

### Quantitative MSP (QMSP) and methylation index (M-index) analyses

We used fluorescence-based real-time PCR for quantitative MSP. The type II collagen gene (*COL2A*) was used as the internal reference gene. Multiplex QMSP was performed in the TaqMan probe system using the LightCycler 480 (Roche Applied Sciences, Mannheim, Germany). The PCR primers and their optimal annealing temperatures are listed in table S3. The 20 µL reaction contained 2 µL of modified template DNA, 1 µL of 20X Custom TaqMan reagent, and 10 µL of LightCycler® 480 Probes Master (Roche Applied Sciences, Mannheim, Germany). In brief, the PCR primers flanked an oligonucleotide probe with a 5′ fluorescent reporter dye (FAM for the target gene and VIC for the reference gene) and a 3′ quencher dye (MGB; Applied Biosystems, Carlsbad, USA). The reactions were subjected to an initial incubation at 95°C for 10 min, followed by 50 cycles of denaturation at 95°C for 10 s, and annealing and extension at 60°C for 40 s. The DNA methylation level was assessed by the methylation index (M-index), using the formula: 10,000×2 ^[(Cp of COL2A) – (Cp of Gene)]^ [26], [28]. Results with Cp values of COL2A greater than 36 were defined as detection failures.

### Immunohistochemical analysis on cervical tissue microarray

The paraffin-embedded cervical tissues of a tissue microarray were prepared from Chinese patients. They were retrieved from the Department of Pathology, Tri-Service General Hospital, and prepared according to a previously published method [29]. The tissue microarray contained 24 samples of normal squamous epithelium, 15 samples of CIN1, 7 samples of CIN2, 16 samples of CIN3/CIS, 58 samples of SCC, and 7 samples of lymph-node-metastatic SCC. The immunohistochemistry procedure followed a standard protocol, using a mouse polyclonal anti-human ZNF582 antibody (H00147948-B01, Abnova, Taiwan) [30]. All tissue microarray slides were examined and scored by two pathologists.

### Statistical analysis

The correlation between categorical variables was determined with Fisher's exact test. Nonparametric statistics such as Kruskal–Wallis rank sum test and Mann–Whitney U test were used to analyze the correlation of continuous variables. The two-sided P-value <0.01 was considered statistically significant. We evaluated the performance of M-index to distinguish diseased samples from control samples by calculating the area under the receiver operating characteristic (ROC) curve (AUC). The statistical power required to distinguish the disease group from the control group was calculated for different cutoff values of the M-index, with an accepted type 1 error of 5% (α = 0.05, two-sided). All analyses were calculated by the statistical package R (R version 2.11.1).

## Results

### Genome-wide promoter methylation analysis and verification of candidate genes in the discovery phase

**Figure 1:**
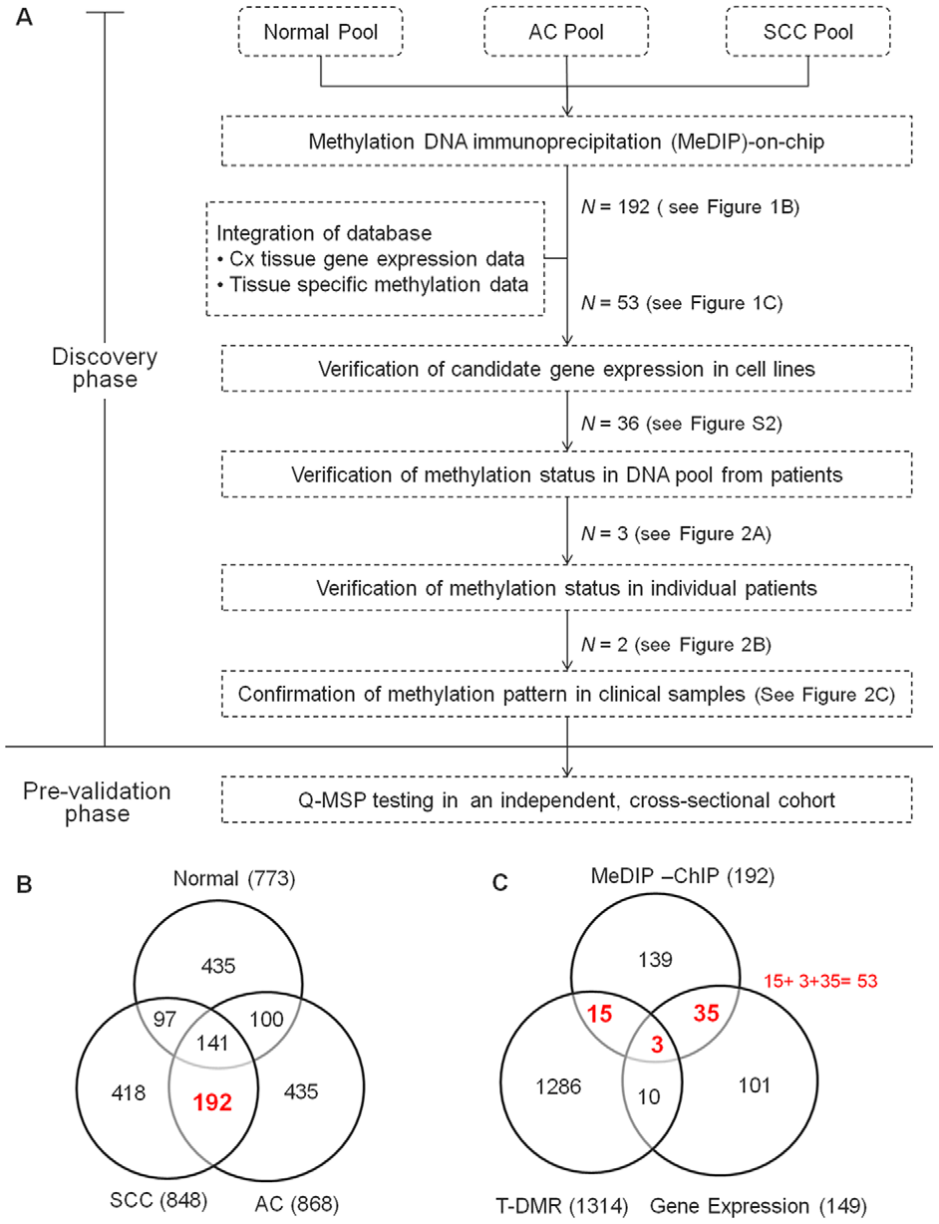
Logistics of the discovery of novel genes epigenetically silenced in cervical cancer using MeDIP-on-chip. (A) Flow chart of the experimental design. *N* means numbers of hypermethylated genes. SCC, Squamous cervical carcinoma; AC, Adenocarcinoma. (B) The Venn diagram shows the number of hypermethylated genes in SCC tissues, AC tissues, and normal cervical cell scrapings. Methylation enrichment occurred in 192 genes for both SCC and AC, but not normal cervixes. (C) The Venn diagram illustrates the integration of MeDIP-on-chip results with gene expression databases and tissue differential methylation regions (T-DMR) data. Genes with mRNA expression greater than 1.2 folds in normal tissues relative to cancer (*N* = 149), and genes within T-DMR (*N* = 1314) were included in the analysis [32].

**Figure 2:**
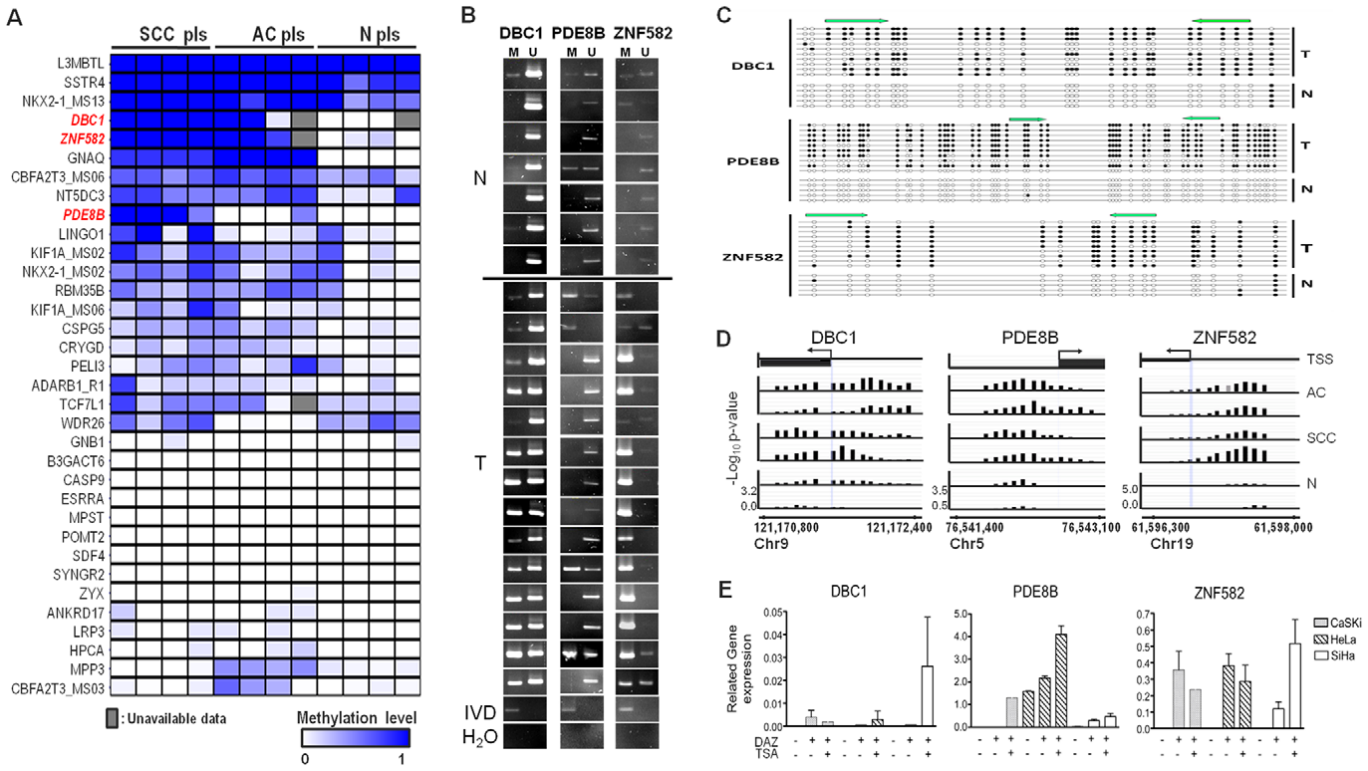
Confirmation of methylation status of promoters in cell lines and clinical samples. (A) Illustrative methylation status of 31 genes, including 34 regions, in pooled normal (N pls), squamous cervical carcinoma (SCC pls), and adenocarcinoma (AC pls) samples detected by of methylation-specific PCR (MSP). Each pool contained DNA from five patients. The bluescale represents the semi-quantitative methylation levels. The gray boxes indicate unavailable results. (B) MSP results for *DBC1*, *PDE8B*, and *ZNF582* in selected individual normal (N) and SCC tissues (T). M: methylation-specific primers; U: nonmethylation-specific primers. *In vitro* methylated DNA (IVD) was used as the positive control. (C) Bisulfite sequencing of *DBC1*, *PED8B*, and *ZNF582* in tumor and normal samples. Each line indicates a single clone. Black and white circles indicate methylated and unmethylated CpG sites, respectively. The green arrows indicate the annealing regions of MSP primers. (D) The location and intensity of probes by MeDIP-on-chip. TSS represents the transcriptional start site and the arrows indicate the direction of mRNA transcription. The Y-axis shows the value from the transforming *P-*value (–log10) by the KS test for each probe. (E) Gene expression analysis after demethylation treatment of cervical cancer cell lines. DAZ, 5-aza-2′-deoxycytidine; TSA, trichostatin A.

The logistics of the study is summarized in Figure 1A. We determined the promoter methylation profiles of uterine cervix using tumor tissues and normal exfoliated cervical cells. The number of methylation enrichment in SCC, AC, and normal cervixes was identified (Figure 1B). We intended to filter the methylation enrichment occurring in both SCC and AC, but not in normal cervixes. The 192 enriched methylation regions at promoters of coding genes were identified and summarized in Table S2. To narrow the candidate list, we integrated these 192 DNA methylation results with candidates from public gene expression and methylation databases (Figure 1C). The public gene expression data, GSE7803, derived from normal cervical, precancerous, and carcinoma tissues, revealed 149 genes with lower levels of gene expression in SCC than in normal cervixes, which is supportive to the methylation-mediated silence concept in cancer [31]. Since the methylation changes in cancer may be similar to those in tissue differentiation, a methylation microarray data set containing 1314 tissue-specific DNA methylation (T-DMR) genes was included in the analysis [32]. Genes, observed in our results and either of these two databases (GSE7803 and T-DMR), were selected for further validation (*N* = 53). The reexpression of these 53 genes treated by demethylating agents in cervical cancer cell lines was assessed. Thirty six genes were confirmed (Figures S2 and 2E). The methylation status of these 34 regions of 31 genes was verified in a small scale of pooled and individual clinical samples by MSP, respectively (Figure 2A and B). Five genes were excluded due to the failure of MSP. Each pool contained equal amounts of DNA from five patients. Nine genes (*L3MBTL*, *SSTR4*, *NKX2-1*, *CBFA2T3, NT5DC3, LINGO1, KIF1A and RBM35B*) methylated in normal samples or blood cells (*GNAQ*, data not shown) were excluded from further analysis (Figure 2A). *DBC1*, *ZNF582*, and *PDE8B* demonstrated high methylation levels in more than four cancer pools, and were chosen for further validation in individual samples.

MSP and bisulfite sequencing were used to evaluate the methylation status of *DBC1*, *PDE8B*, and *ZNF582* in a small-scaled individual sample. In tumor tissues, the methylation frequencies of *DBC1*, *PDE8B*, and *ZNF582* were 13/14 (93%), 4/14 (29%), and 14/14 (100%), respectively (Figure 2B). Bisulfite sequencing of these genes confirmed the hypermethylation status in cervical cancers (Figure 2C). The location and intensity of probes by MeDIP-on-chip are shown in Figure 2D. Demethylation treatment restored the expression of these genes in cervical cancer cell lines (Figure 2E). *DBC1* and *ZNF582* were selected for testing in an independent clinical cohort.

### Validation by quantitative methylation analysis in an independent, cross-sectional cohort

**Figure 3:**
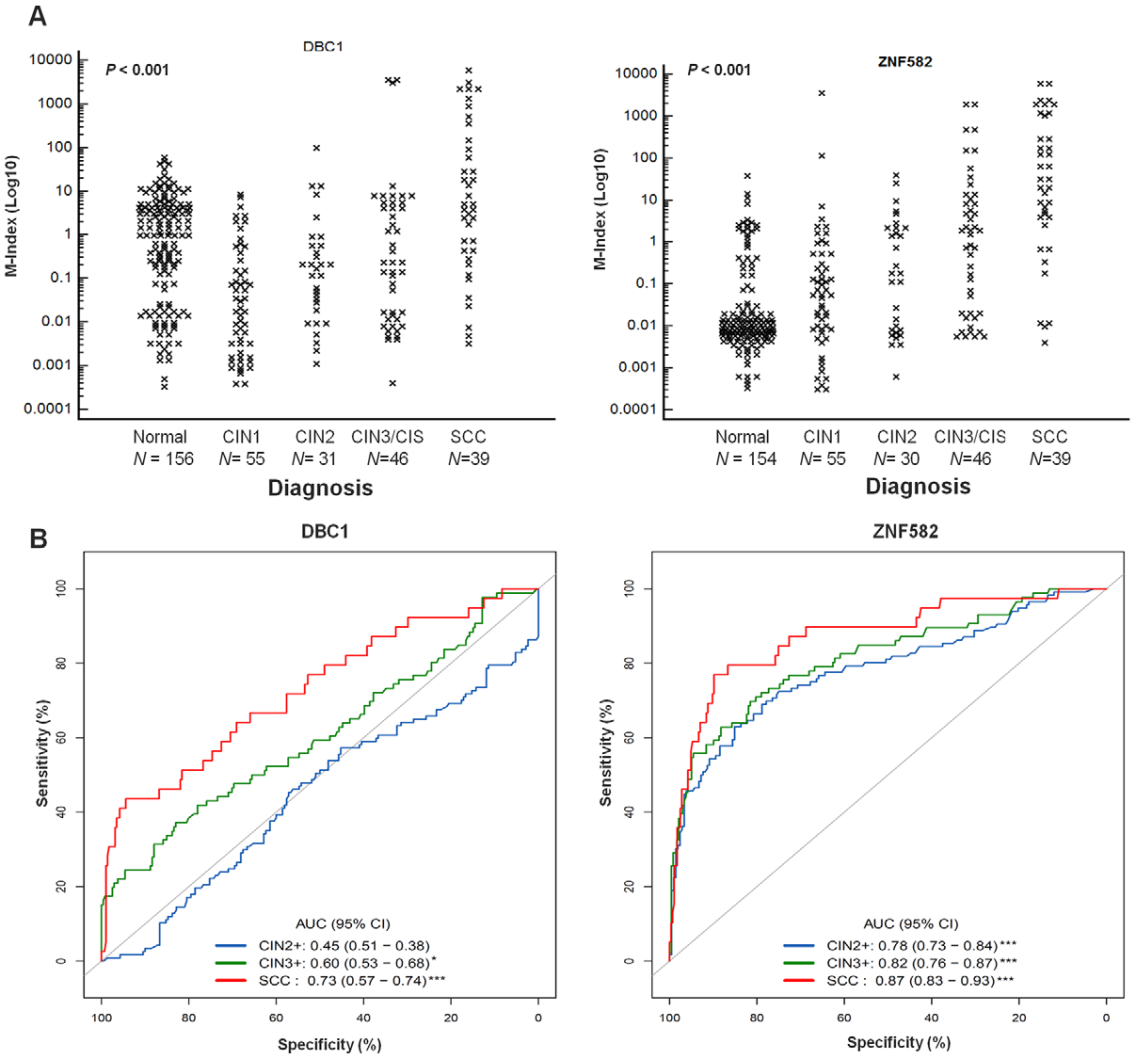
QMSP analysis of *DBC1* and *ZNF582* in cervical scrapings. (A) Dot plots illustrate the M-index distributions. *N* represents the case number. *P-*value <0.001 was determined by the nonparametric Kruskal–Wallis rank sum test. (B) The areas under the receiver operating curves (AUCs) used to estimate accuracy. (*P-value <0.05, ***P-value <0.001; CI, confidence interval).

To further validate the clinical utility of these genes, the methylation status of *DBC1* and *ZNF582* in cervical scrapings, rather than in the tissues, was tested using QMSP. The M-indexes for *DBC* and *ZNF582* showed significant increasing trends with worsening cervical lesions (*P*<0.001; Figure 3A). Table 1 showed the median M-index for each disease category. The median M-index for *DBC1* was 5.11 in SCC, which is significantly higher than that in normal controls (median  = 1.20; *P*<0.001). The median M-indexes for *ZNF582* in CIN2, CIN3/CIS, and SCC were 0.19, 1.71, and 31.95, respectively, and all were significantly higher than those in normal controls. When the diagnoses were dichotomized as CIN2+ and CIN1^–^ (including CIN1 and normal cervix), the M-index for *ZNF582* can significantly distinguish these two groups (*P*<0.001, Table 2). *ZNF582* methylation had accuracies of 0.78, 0.82, and 0.87 in the detection of CIN2+, CIN3+, and SCC lesions, respectively (Figure 3B). Cutoff values of M-index for the detection of CIN2+, CIN3+ or SCC were assessed (Table 2). At a cutoff value of 0.62, the sensitivity and specificity of *ZNF582* in the detection of CIN3+ were 0.70 and 0.82, respectively, with an accuracy of 0.80.

**Table 1:** The Methylation Index (M-Index) of DBC1 and ZNF582 in the spectrum of cervical lesions.

		DBC1***		ZNF582***	
Diagnosis	Case Number	Median	95% CI	Median	95% CI
Normal	156	1.20	0.522 – 2.292	0.01	0.008 – 0.013
CIN1	55	0.02	0.006 – 0.082	0.06	0.017 – 0.141
CIN2	31	0.17	0.034 – 0.432	0.19	0.009 – 1.973†
CIN3/CIS	46	0.24	0.020 – 2.547	1.71	0.135 – 4.747‡
SCC	39	5.11	0.898 – 30.035‡	31.95	5.653 – 171.926‡

Abbreviation: CI, confidence interval; CIN1, cervical intraepithelial neoplasia type 1, CIN2, cervical intraepithelial neoplasia type 2; CIN3, cervical intraepithelial neoplasia type 3; CIS, carcinoma *in situ*; SCC, squamous cervical carcinoma. DBC1, deleted DNA in bladder cancer 1; ZNF582, zinc finger 582.

*** *P*-value <0.001 obtained using Kruskal-Wallis rank sum test.

† *P*-value  = 0.002 compared with normal and obtained using Mann-Whitney U test.

‡ *P*-value <0.001 compared with normal and obtained using Mann-Whitney U test.

**Table 2:** The area under the ROC curve analysis for distinguishing different diagnosis groups.

Gene Name	Case/Control	Cutoff value	AUC	(95% CI)	Sensitivity	(95% CI)	Specificity	(95% CI)
DBC1	CIN2+/CIN1−	0.97	0.45	(0.51–0.38)	0.57	(0.48–0.66)	0.45	(0.39–0.52)
	CIN3+/CIN2−	5.09	0.60	(0.53–0.68)†	0.37	(0.27–0.48)	0.83	(0.78–0.88)
	SCC/SCC−	13.60	0.73	(0.57–0.74)‡	0.44	(0.28–0.59)	0.94	(0.92–0.97)
ZNF582	CIN2+/CIN1−	0.62	0.78	(0.73–0.84)‡	0.63	(0.54–0.72)	0.85	(0.80–0.90)
	CIN3+/CIN2−	0.62	0.82	(0.76–0.87)‡	0.70	(0.60–0.79)	0.82	(0.76–0.87)
	SCC/SCC−	3.77	0.87	(0.83–0.93)‡	0.77	(0.64–0.90)	0.90	(0.86–0.93)

Abbreviation: ROC, the receiver operation characteristics; AUC, the area under the ROC curve; CI, confidence interval; CIN2, cervical intraepithelial neoplasia type 2; CIN3, cervical intraepithelial neoplasia type 3; SCC, squamous cervical carcinoma; +, the descriptive and worse diagnosis; −, the better diagnosis. DBC1, deleted DNA in bladder cancer 1; ZNF582, Krüppel-type zinc finger 582.

† *P*-value <0.01 obtained using the Mann-Whitney U test.

‡ *P*-value <0.001 obtained using the Mann-Whitney U test.

### ZNF582 protein is expressed in human precancerous cervical lesions, but not in invasive cancer

**Figure 4:**
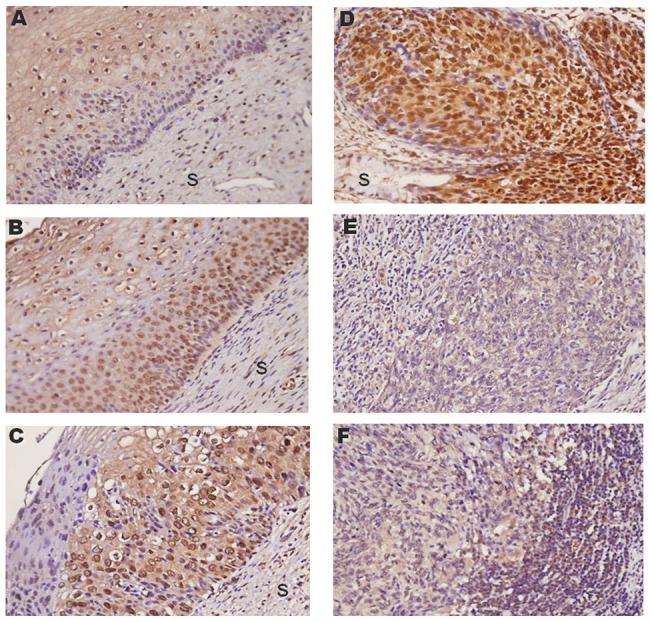
Representative expression of ZNF582 protein in normal and abnormal cervical tissues. (A) Normal squamous epithelium *(N* = 24). (B) and (C), Cervical intraepithelial neoplasias 1 and 2 (CIN1 and CIN2, *N* = 22). (D) Carcinoma *in situ* (CIS, *N* = 16). (E) and (F), Squamous cervical carcinoma (SCC, *N* = 58) and metastatic forms (*N* = 7), respectively (x400). S, stroma.

Immunohistochemical staining for ZNF582 in a cervical tissue microarray containing 127 samples from patients with different disease statues revealed the protein expression of ZNF582 in real human tissues (Figure 4). ZNF582 protein expression increased significantly from normal squamous epithelium to CIS, and then decreased as the cancer cells progressed to invasive and metastatic forms. These results supported the increasingly frequent DNA methylation detected by a PCR-based method.

## Discussion

Attempts to detect cervical cancer using DNA methylation have been hampered by the lack of consistent results for methylation analyses. The sensitivity and specificity afforded by most published genes are moderate and not clinically applicable yet [7]. Recent studies, including ours, using quantitative methylation analyses have shown that DNA methylation is a potential biomarker for improved screening [24], [33], and for the triage of mildly abnormal Pap smears [34] or high-risk HPV-positive women [35]–[38]. New genes displaying cancer-associated DNA methylation must be identified to improve the performance of a DNA methylation biomarker panel. In this study, we first used MeDIP-on-chip to profile the methylation status of genes in cervical clinical samples. This method has proved powerful in previous methylomic analyses [39], [40]. However, the application of this technology to clinical cancer tissues is limited [41], [42]. The advantage of using clinical samples is to reduce the overestimation of hypermethylation in cell line, that can not be verified in clinical samples [43]. We used pooled DNA that adjusts individual variations and gets more common instances of DNA methylation biomarkers. Newly identified DNA methylation in cervical cancer tissues will not be useful for screening unless this methylation can be detected in clinically accessible materials such as the cervical scrapings. We identified candidate genes in cancer tissues, narrowed the candidate list, and confirmed the most likely genes using QMSP in cervical scrapings from a full spectrum of cervical neoplasms. The present study has identified, for the first time, the hypermethylated gene, *ZNF582*, with possible utility in the molecular detection of cervical cancer.

*ZNF582*, located at chromosome 19q13.43, encodes the Krüppel-type zinc finger protein 582 (HGNC: 26421), which contains one KRAB-A-B domain and nine zinc-finger motifs [44]. A recent study of acute myeloid leukemia revealed that *ZNF582* is consistently aberrantly methylated in different disease subtypes [45]. However, the biological function of ZNF582 is not yet well characterized. Most KRAB-ZNF proteins contain the KRAB (AB) domain and bind KRAB-associated protein 1 (KAP1) to corepress gene transcription [46], [47]. Members of the KRAB-ZNF family are probably involved in a variety of biological processes related to the DNA damage response, proliferation, cell cycle control, and neoplastic transformation [46]. The present study found that *ZNF582* is highly methylated in invasive cancer tissues. Although protein expression in carcinoma *in situ* is high, we detected the DNA methylation of *ZNF582*, that indicated the molecular propensity of some *in situ* cells toward cancer invasion. The expression of ZNF582 in precancerous lesions in accordance with disease severity in tissue sections may fail to support the role of *ZNF582* as a tumor suppressor gene. However, its silencing in invasive cancer lesions suggests that *ZNF582* may be a tumor suppressor; the expression of which increases in response to oncogenic stress in precancer stages. Further investigation of the functional role of ZNF582 in cervical cancer may provide more biological insight in cancer biology.

In contrast, *DBC1*, located at 9q32–33, is reported to display frequent loss of heterozygosity in bladder cancers [48], lymphoproliferative malignancies [49], and non-small-cell lung cancers [50], [51]. The overexpression of DBC1 increased cell death in cultured bladder cancer cell lines and inhibited cell growth of non-small-cell lung cancer cell lines [51], [52]. These reports suggest that DBC1 has a tumor-suppressive role.

The ideal DNA methylation biomarker panel, which fulfills the various requirements for cervical cancer detection within different infrastructures, has yet to be established. In previous studies, we discovered that *SOX1*, *NKX6-1*, *PAX1*, *WT1*, and *LMX1A* are highly methylated in cervical cancer [22]. A quantitative methylation analysis of these genes showed sensitivities in the range of 0.77–0.93 and specificities of 0.82–0.97 for CIN3+ lesions in a single-hospital-based cross-sectional setting [24]. Previous results also demonstrated the potential utility of quantitative *PAX1* methylation in the triage of patients with mildly abnormal Pap smears for the prediction of high-grade lesions, with a sensitivity of 0.88 and specificity of 0.98 [34]. These encouraging results have been subjected to an ongoing multicenter validation study in Taiwan. The latest report of quantitative methylation biomarkers for the triage of high-risk HPV-positive women revealed that the combined methylation analysis of *CADM1* and *MAL* distinguished CIN3+ lesions as effectively as cytology (sensitivity and specificity of 0.66 and 0.79, respectively) or cytology/HPV genotyping (sensitivity and specificity of 0.84 and 0.54, respectively) [38]. The AUC of this combination was 0.72. In the present study, *ZNF582* methylation alone conferred an AUC of 0.82, with a sensitivity of 0.70 and specificity of 0.82, which is equivalent to the performance of cytology. A standardized assay and population-based studies are required to evaluate the usefulness of this gene in molecular cervical cancer screening. However, a standardized assay and population-based study are required to evaluate the usefulness of this gene in molecular cervical cancer screening.

A DNA methylation biomarker as effective as the conventional Pap smear should be sufficient for women in developing countries lacking a cytology-based infrastructure. Combined testing with DNA methylation biomarkers and cytology may improve the unsatisfactory sensitivity of cytology alone without seriously compromising its specificity, or it may help in the triage of mildly abnormal Pap smears in developed countries where the cytology infrastructure has reached its limits. These proposed applications of DNA methylation in cervical cancer warrant further investigations.

Technological advances may facilitate the discovery of novel instances of cancer-specific DNA methylation and their translation to clinical diagnostics. DMH and RLGS have been used for this purpose, but they have been limited by the available enzymatic cutting sites [7], [22], [42]. Some investigators used bisulfite conversed DNA to apply to genome-wide methylation profiling by bead array platforms [53]. Further improvement of immunoprecipitation using anti-5-methylcytosine or anti-methyl-CpG-binding domain antibody coupled with next-generation sequencing technologies may provide a more comprehensive methylomic analysis [54]. It may improve the performance of the cervical cancer methylation panel.

The standardization and quality control of methylation testing is also important in the development of these biomarkers for clinical diagnostics. QMSP is fast and sensitive in quantifying the methylation status of specific genes, and can be used to screen many clinical samples simultaneously [5], [26], [55]. The optimal testing condition for clinical purposes is necessary. Genetic polymorphisms at the target sequences may affect the results of QMSP. According to dbSNP build 130 database, there is a single nucleotide polymorphism (SNP) (rs11791877, C/G) in the forward primer of *DBC1*. This may be one of the reasons that its accuracy is compromised in the testing of clinical samples. There are no SNPs in primers and probe for *ZNF582*.

In conclusion, we identified *ZNF582* as a potential candidate gene in the development of a novel strategy for molecular cervical cancer screening. The discovery and translation of new DNA methylation biomarkers may be a useful tool in the screening of cervical cancer in the near future.

## Supporting Information

Figure S1 The intensity of probes by MeDIP-on-chip for thirty-two regions (including 35 genes) in cervical tissue.
(TIF)

Figure S2 Genes re-expression analysis by QRT-PCR in cervical cell lines.
(TIF)

Table S1 Demographic for discovery and pre-validation phase studies.
(PDF)

Table S2 Selected 192 methylated genes in SCC and AC.
(PDF)

Table S3 The summary of polymerase chain reaction primers.
(PDF)

Material and Methods S1 (DOC)

## References

[1] Forouzanfar MH, Foreman KJ, Delossantos AM, Lozano R, Lopez AD (2011). Breast and cervical cancer in 187 countries between 1980 and 2010: a systematic analysis.. Lancet.

[2] Walboomers JM, Jacobs MV, Manos MM, Bosch FX, Kummer JA (1999). Human papillomavirus is a necessary cause of invasive cervical cancer worldwide.. J Pathol.

[3] Woodman CB, Collins SI, Young LS (2007). The natural history of cervical HPV infection: unresolved issues.. Nat Rev Cancer.

[4] Brinton LA, Hamman RF, Huggins GR, Lehman HF, Levine RS (1987). Sexual and reproductive risk factors for invasive squamous cell cervical cancer.. J Natl Cancer Inst.

[5] Cottrell SE, Laird PW (2003). Sensitive detection of DNA methylation.. Ann N Y Acad Sci.

[6] Hellberg D, Stendahl U (2005). The biological role of smoking, oral contraceptive use and endogenous sexual steroid hormones in invasive squamous epithelial cervical cancer.. Anticancer Res.

[7] Wentzensen N, Sherman ME, Schiffman M, Wang SS (2009). Utility of methylation markers in cervical cancer early detection: appraisal of the state-of-the-science.. Gynecol Oncol.

[8] Ziegler RG, Weinstein SJ, Fears TR (2002). Nutritional and genetic inefficiencies in one-carbon metabolism and cervical cancer risk.. J Nutr.

[9] Nanda K, McCrory DC, Myers ER, Bastian LA, Hasselblad V (2000). Accuracy of the Papanicolaou test in screening for and follow-up of cervical cytologic abnormalities: a systematic review.. Ann Intern Med.

[10] Lynge E, Rebolj M (2009). Primary HPV screening for cervical cancer prevention: results from European trials.. Nat Rev Clin Oncol.

[11] Kulasingam SL, Hughes JP, Kiviat NB, Mao C, Weiss NS (2002). Evaluation of human papillomavirus testing in primary screening for cervical abnormalities: comparison of sensitivity, specificity, and frequency of referral.. JAMA.

[12] Chaturvedi AK, Engels EA, Pfeiffer RM, Hernandez BY, Xiao W (2011). Human papillomavirus and rising oropharyngeal cancer incidence in the United States.. J Clin Oncol.

[13] Koliopoulos G, Chrelias C, Pappas A, Makridima S, Kountouris E (2012). The diagnostic accuracy of two methods for E6&7 mRNA detection in women with minor cytological abnormalities.. Acta Obstet Gynecol Scand.

[14] Negri G, Bellisano G, Carico E, Faa G, Kasal A (2011). Usefulness of p16ink4a, ProEX C, and Ki-67 for the diagnosis of glandular dysplasia and adenocarcinoma of the cervix uteri.. Int J Gynecol Pathol.

[15] Schiffman M, Wentzensen N, Wacholder S, Kinney W, Gage JC (2011). Human papillomavirus testing in the prevention of cervical cancer.. J Natl Cancer Inst.

[16] Silling S, Kreuter A, Hellmich M, Swoboda J, Pfister H (2012). Human papillomavirus oncogene mRNA testing for the detection of anal dysplasia in HIV-positive men who have sex with men.. J Clin Virol.

[17] Sorbye SW, Fismen S, Gutteberg T, Mortensen ES (2010). Triage of women with minor cervical lesions: data suggesting a “test and treat” approach for HPV E6/E7 mRNA testing.. PLoS One.

[18] Tsimplaki E, Argyri E, Michala L, Kouvousi M, Apostolaki A (2012). Human papillomavirus genotyping and e6/e7 mRNA expression in greek women with intraepithelial neoplasia and squamous cell carcinoma of the vagina and vulva.. J Oncol.

[19] Bird A (2002). DNA methylation patterns and epigenetic memory.. Genes Dev.

[20] Sharma S, Kelly TK, Jones PA (2010). Epigenetics in cancer.. Carcinogenesis.

[21] Laird PW (2003). The power and the promise of DNA methylation markers.. Nat Rev Cancer.

[22] Lai HC, Lin YW, Huang TH, Yan P, Huang RL (2008). Identification of novel DNA methylation markers in cervical cancer.. Int J Cancer.

[23] Wang SS, Smiraglia DJ, Wu YZ, Ghosh S, Rader JS (2008). Identification of novel methylation markers in cervical cancer using restriction landmark genomic scanning.. Cancer Res.

[24] Lai HC, Lin YW, Huang RL, Chung MT, Wang HC (2010). Quantitative DNA methylation analysis detects cervical intraepithelial neoplasms type 3 and worse.. Cancer.

[25] Sova P, Feng Q, Geiss G, Wood T, Strauss R (2006). Discovery of novel methylation biomarkers in cervical carcinoma by global demethylation and microarray analysis.. Cancer Epidemiol Biomarkers Prev.

[26] Eads CA, Danenberg KD, Kawakami K, Saltz LB, Blake C (2000). MethyLight: a high-throughput assay to measure DNA methylation.. Nucleic Acids Res.

[27] Lai HC, Lin YW, Chang CC, Wang HC, Chu TW (2007). Hypermethylation of two consecutive tumor suppressor genes, BLU and RASSF1A, located at 3p21.3 in cervical neoplasias.. Gynecol Oncol.

[28] Reesink-Peters N, Wisman GB, Jeronimo C, Tokumaru CY, Cohen Y (2004). Detecting cervical cancer by quantitative promoter hypermethylation assay on cervical scrapings: a feasibility study.. Mol Cancer Res.

[29] Seo HS, Liu DD, Bekele BN, Kim MK, Pisters K (2008). Cyclic AMP response element-binding protein overexpression: a feature associated with negative prognosis in never smokers with non-small cell lung cancer.. Cancer Res.

[30] Liu CY, Chao TK, Su PH, Lee HY, Shih YL (2009). Characterization of LMX-1A as a metastasis suppressor in cervical cancer.. J Pathol.

[31] Zhai Y, Kuick R, Nan B, Ota I, Weiss SJ (2007). Gene expression analysis of preinvasive and invasive cervical squamous cell carcinomas identifies HOXC10 as a key mediator of invasion.. Cancer Res.

[32] Irizarry RA, Ladd-Acosta C, Wen B, Wu Z, Montano C (2009). The human colon cancer methylome shows similar hypo- and hypermethylation at conserved tissue-specific CpG island shores.. Nat Genet.

[33] Lim EH, Ng SL, Li JL, Chang AR, Ng J (2010). Cervical dysplasia: assessing methylation status (Methylight) of CCNA1, DAPK1, HS3ST2, PAX1 and TFPI2 to improve diagnostic accuracy.. Gynecol Oncol.

[34] Chao TK, Ke FY, Liao YP, Wang HC, Yu CP (2011). Triage of cervical cytological diagnoses of atypical squamous cells by DNA methylation of paired boxed gene 1 (PAX1).. Diagn Cytopathol.

[35] Overmeer RM, Louwers JA, Meijer CJ, van Kemenade FJ, Hesselink AT (2011). Combined CADM1 and MAL promoter methylation analysis to detect (pre-)malignant cervical lesions in high-risk HPV-positive women.. Int J Cancer.

[36] van der Meide WF, Snellenberg S, Meijer CJ, Baalbergen A, Helmerhorst TJ (2011). Promoter methylation analysis of WNT/beta-catenin signaling pathway regulators to detect adenocarcinoma or its precursor lesion of the cervix.. Gynecol Oncol.

[37] Eijsink JJ, Lendvai A, Deregowski V, Klip HG, Verpooten G (2011). A four gene methylation marker panel as triage test in hr-HPV positive patients.. Int J Cancer.

[38] Hesselink AT, Heideman DA, Steenbergen RD, Coupe VM, Overmeer RM (2011). Combined promoter methylation analysis of CADM1 and MAL: an objective triage tool for high-risk human papillomavirus DNA-positive women.. Clin Cancer Res.

[39] Weber M, Davies JJ, Wittig D, Oakeley EJ, Haase M (2005). Chromosome-wide and promoter-specific analyses identify sites of differential DNA methylation in normal and transformed human cells.. Nat Genet.

[40] Feinberg AP (2010). Genome-scale approaches to the epigenetics of common human disease.. Virchows Arch.

[41] Jacinto FV, Ballestar E, Ropero S, Esteller M (2007). Discovery of epigenetically silenced genes by methylated DNA immunoprecipitation in colon cancer cells.. Cancer Res.

[42] Cheung HH, Lee TL, Davis AJ, Taft DH, Rennert OM (2010). Genome-wide DNA methylation profiling reveals novel epigenetically regulated genes and non-coding RNAs in human testicular cancer.. Br J Cancer.

[43] Smiraglia DJ, Rush LJ, Fruhwald MC, Dai Z, Held WA (2001). Excessive CpG island hypermethylation in cancer cell lines versus primary human malignancies.. Hum Mol Genet.

[44] Huntley S, Baggott DM, Hamilton AT, Tran-Gyamfi M, Yang S (2006). A comprehensive catalog of human KRAB-associated zinc finger genes: insights into the evolutionary history of a large family of transcriptional repressors.. Genome Res.

[45] Figueroa ME, Lugthart S, Li Y, Erpelinck-Verschueren C, Deng X (2010). DNA Methylation Signatures Identify Biologically Distinct Subtypes in Acute Myeloid Leukemia.. Cancer Cell.

[46] Urrutia R (2003). KRAB-containing zinc-finger repressor proteins.. Genome Biol.

[47] Peng H, Gibson LC, Capili AD, Borden KL, Osborne MJ (2007). The structurally disordered KRAB repression domain is incorporated into a protease resistant core upon binding to KAP-1-RBCC domain.. J Mol Biol.

[48] Habuchi T, Luscombe M, Elder PA, Knowles MA (1998). Structure and methylation-based silencing of a gene (DBCCR1) within a candidate bladder cancer tumor suppressor region at 9q32-q33.. Genomics.

[49] Gronbaek K, Ralfkiaer U, Dahl C, Hother C, Burns JS (2008). Frequent hypermethylation of DBC1 in malignant lymphoproliferative neoplasms.. Mod Pathol.

[50] Habuchi T, Takahashi T, Kakinuma H, Wang L, Tsuchiya N (2001). Hypermethylation at 9q32-33 tumour suppressor region is age-related in normal urothelium and an early and frequent alteration in bladder cancer.. Oncogene.

[51] Izumi H, Inoue J, Yokoi S, Hosoda H, Shibata T (2005). Frequent silencing of DBC1 is by genetic or epigenetic mechanisms in non-small cell lung cancers.. Hum Mol Genet.

[52] Wright KO, Messing EM, Reeder JE (2004). DBCCR1 mediates death in cultured bladder tumor cells.. Oncogene.

[53] Bibikova M, Fan J–B (2010). Genome-wide DNA methylation profiling.. Wiley Interdisciplinary Reviews: Systems Biology and Medicine.

[54] Laird PW (2010). Principles and challenges of genome-wide DNA methylation analysis.. Nat Rev Genet.

[55] Cottrell SE (2004). Molecular diagnostic applications of DNA methylation technology.. Clin Biochem.

