# Supplementary figures and images for: Methylomic Analysis Identifies Frequent DNA Methylation of Zinc Finger Protein 582 (ZNF582) in Cervical Neoplasms
Source: PLoS One. 2012 Jul 16;7(7):e41060. doi: 10.1371/journal.pone.0041060 (PMC3397950; doi:10.1371/journal.pone.0041060)

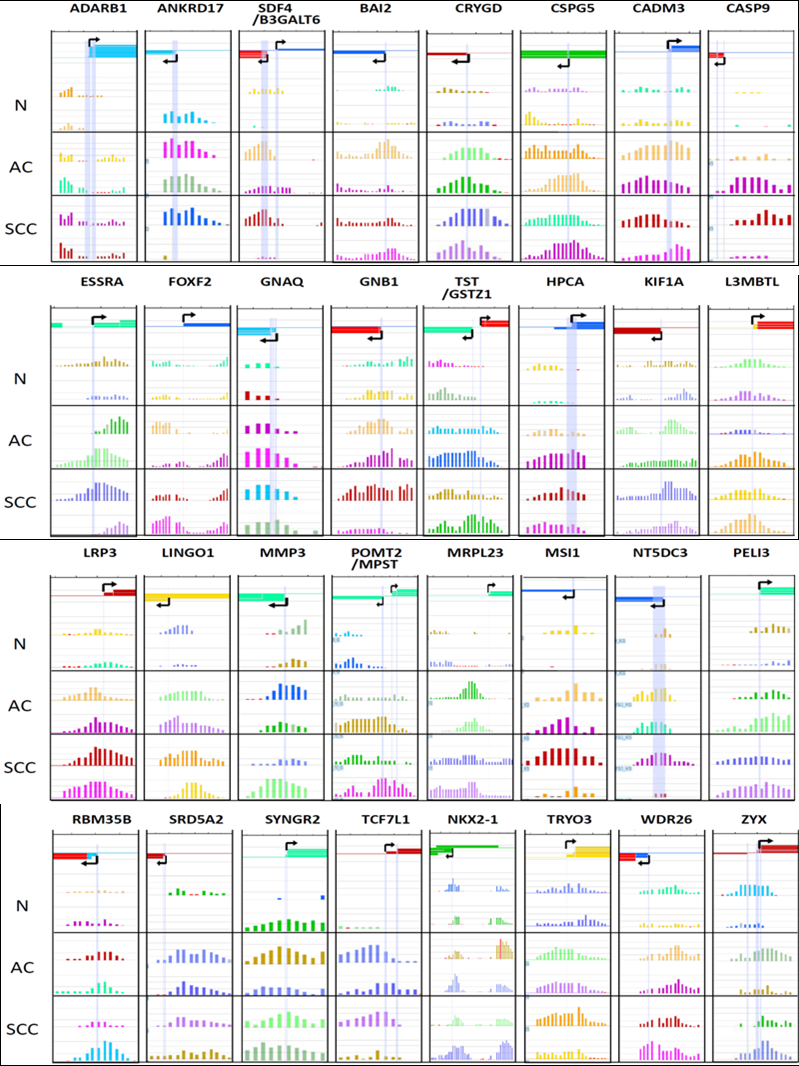

Supplement: Figure S1 — The intensity of probes by MeDIP-on-chip for thirty-two regions (including 35 genes) in cervical tissue. (TIF) [file pone.0041060.s001.tif]

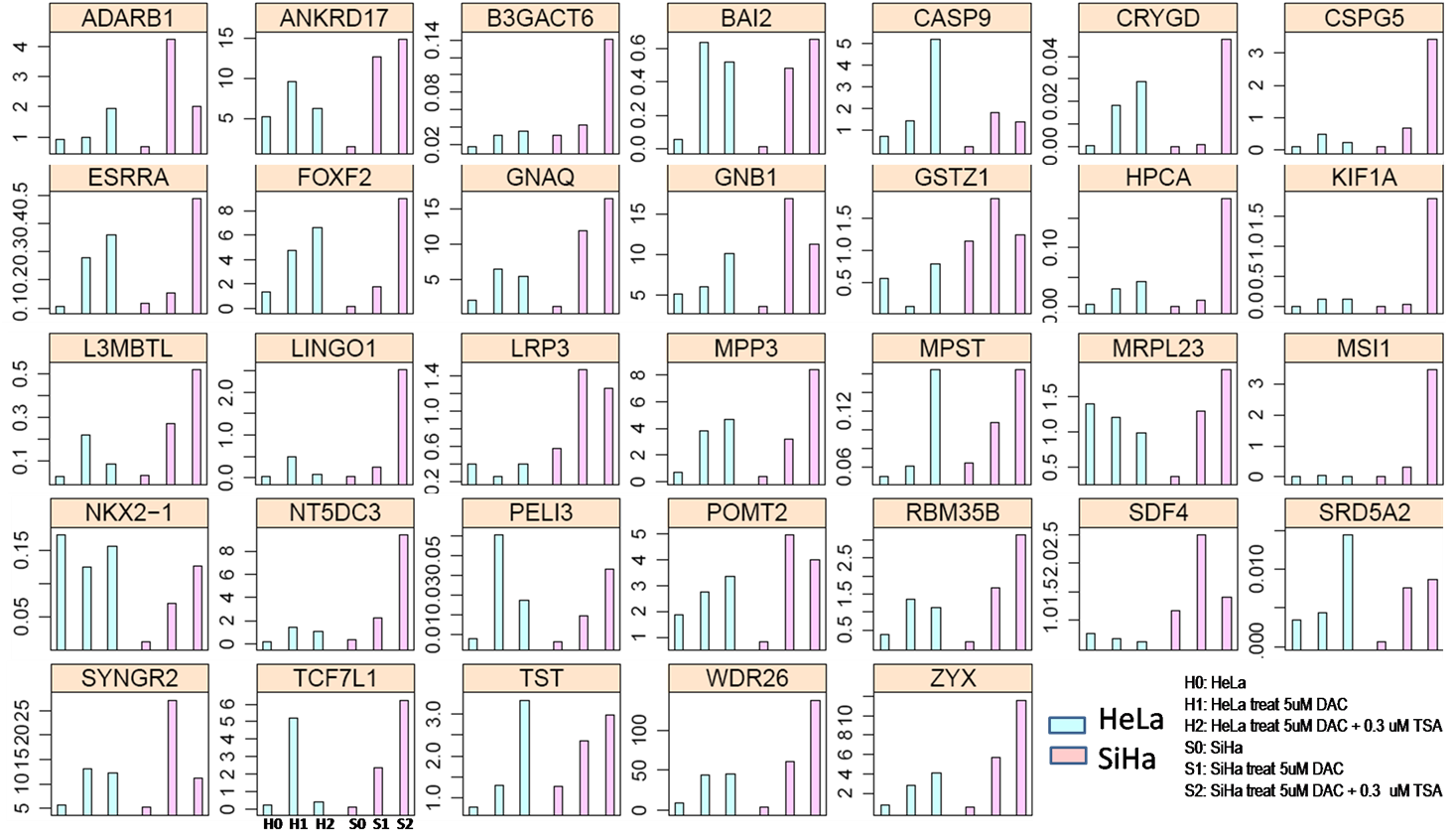

Supplement: Figure S2 — Genes re-expression analysis by QRT-PCR in cervical cell lines. (TIF) [file pone.0041060.s002.tif]
