# Supplementary material for: Methylomic Analysis Identifies Frequent DNA Methylation of Zinc Finger Protein 582 (ZNF582) in Cervical Neoplasms
Source: PLoS One. 2012 Jul 16;7(7):e41060. doi: 10.1371/journal.pone.0041060 (PMC3397950; doi:10.1371/journal.pone.0041060)
Supplement: Table S1 — Demographic for discovery and pre-validation phase studies. (PDF) [file pone.0041060.s003.pdf]

Table S1. Demographic for Discovery and pre-validation phase studies

Histopathology	Case Number	Age Mean	±	SD
In discovery phase				
Normal	19	46.5	±	12.67
AC	19	50.7	±	11.19
SCC	38	51.4	±	11.22
Total	76			
In pre-validation phase				
Normal	156	44.4	±	13.71
CIN1	55	42.1	±	9.48
CIN2	31	45.6	±	14.92
CIN3/CIS	47	47.3	±	11.56
SCC	41	55.4	±	13.28
Total	330			

Abbreviations: SD, Standard deviation; Normal, normal scrapings; AC, adenocarcinoma; SCC, squamous cervical carcinoma; CIN1, cervical intraepithelial neoplasia type 1, CIN2, cervical intraepithelial neoplasia type 2; CIN3, cervical intraepithelial neoplasia type 3; CIS, carcinoma *in situ* .
