# Supplementary material for: Methylomic Analysis Identifies Frequent DNA Methylation of Zinc Finger Protein 582 (ZNF582) in Cervical Neoplasms
Source: PLoS One. 2012 Jul 16;7(7):e41060. doi: 10.1371/journal.pone.0041060 (PMC3397950; doi:10.1371/journal.pone.0041060)
Supplement: Table S2 — Selected 192 methylated genes in SCC and AC. (PDF) [file pone.0041060.s004.pdf]

Table S2. Selected 192 methylated genes in SCC and AC

Gene name	Chr	Strand	Refseq	Methylated region in AC	Methylated region in SCC
ADAMTS20	chr12	-	NM_025003	42231794~42232058	42231494~42231958
ADARB1	chr21	+	NM_015834	45318157~45318496	45318157~45318311<br>45318367~45318496
AK001533	chr7	+	AK001533	98447664~98447918	98447759~98448123
ANKRD17	chr4	-	NM_198889	74343190~74343629<br>74343290~74343629	 74343190~74343629
ARHGDIG	chr16	+	NM_001176	270231~270770<br>270406~270855	270106~270680
ASPHD1	chr16	+	NM_181718	29819114~29819674	29819034~29819484
ATP11B	chr3	+	NM_014616	183993987~183994151	183993987~183994151
B3GALT6	chr1	+	NM_080605	1156925~1157265	1156925~1157265
B4GALNT4	chr11	+	NM_178537	359004~359474	359106~359559
BAI2	chr1	-	NM_001703	32002042~32002486	32002257~32002486
C10orf92	chr10	-	NM_017609	134521836~134522385	134522141~134522295
C19orf22	chr19	-	NM_138774	864002~864573	864122~864462
C7orf28B	chr7	-	NM_198097	6832084~6832628	6832084~6832443
CACNG8	chr19	+	NM_031895	59157320~59157774<br>59157620~59157774	59157515~59157774
CADM3	chr1	+	NM_021189	157407348~157408108<br>157407543~157408408	157407543~157407892<br>157408059~157408408
CASP9	chr1	-	NM_001229	15723533~15723982	15723833~15723982
CBFA2T3	chr16	-	NM_175931	87535340~87536001<br>87535655~87536001	 87535140~87535389
CBLN1	chr16	-	BC093692	47873142~47873491	47872962~47873411
CBLN2	chr18	-	BC035789	68361755~68362009	68361070~68361909
CD7	chr17	-	NM_006137	77868872~77869326	77868972~77869326
CGREF1	chr2	-	NM_006569	27195200~27195849	27195610~27195771
CHAD	chr17	-	NM_001267	45901090~45901434	45901090~45901339
CNOT6	chr5	+	NM_015455	179853829~179854093	179853929~179854493
CNOT6L	chr4	-	NM_144571	78959355~78960102	78959545~78960202
COL18A1	chr21	+	NM_130445	45649336~45649878	45648728~45649275
COX8C	chr14	+	NM_182971	92883198~92883942	92883198~92883942
CSPG5	chr3	-	NM_006574	47594935~47595474	47594836~47595474
CTCFL	chr20	-	NM_080618	55533670~55534331<br>55533977~55534136	 55533977~55534231
CTDSPL	chr3	+	NM_005808	37878667~37878916	37878157~37878291
CX36	chr15	-	NM_020660	32834156~32834599	32833860~32834205
CXCL14	chr5	-	NM_004887	134942482~134942836	134942482~134942951
CXorf40B	chrX	-	NM_00101384	148857626~148858170	148858012~148858170<br>148857706~148858170
DNMT3L	chr21	-	NM_175867	44506343~44506878	44506537~44506993
DOCK6	chr19	-	NM_020812	11234164~11234401	11234164~11234306
ECAT11	chr1	+	NM_019079	62433031~62433275	62432531~62433185
EML3	chr11	-	NM_153265	62136237~62136983	62136237~62136983
ESRRA	chr11	+	BC092470	63829442~63830306<br>63830047~63830496	63829442~63830306<br>63830257~63830411
EXD3	chr9	-	FLJ20433	139437582~139438226	139437667~139437931<br>139436867~139437631
F8A1	chrX	+	BC039693<br>NM_012151	 153766132~153767681	154263538~154264501
FAM100A	chr16	-	NM_145253	4604515~4604779	4604515~4604779
FAM91A2	chr1	+	XM_930235	147842673~147842822	147842673~147842822
FLJ36070	chr19	-	AK131427	53914749~53915118	53914664~53915198

Table S2. Selected 192 methylated genes in SCC and AC

Gene name	Chr	Strand	Refseq	Methylated region in AC	Methylated region in SCC
FOXF2	chr6	+	NM_001452	1334254~1334688	1334154~1334989
GNAQ	chr9	-	NM_002072	79835716~79836075	79835826~79836075
					79835716~79836075
GNB1	chr1	-	NM_002074	1812269~1812438	1811990~1812438
				1812389~1812638	1812969~1813128
GRAMD1A	chr19	+	NM_020895	40183007~40183459	40183303~40183534
GSTZ1	chr14	+	NM_001513	76856814~76857059	76856609~76857269
GYG2	chrX	+	U94364	2757470~2757714	2757665~2758019
				2757470~2757914	
HPCA	chr1	+	NM_002143	33124008~33124934	33124008~33124533
					33124189~33124834
HSPG2	chr1	-	NM_005529	22136448~22137106	22136657~22137106
IDI1	chr10	+	BC057827	1092776~1093140	1093091~1093245
IRAK1	chrX	-	NM_001569	152938679~152938838	152938679~152938838
KBTBD9	chr2	+	AL834515	23638368~23638507	23638368~23638507
KIF1A	chr2	-	NM_004321	241408528~241408780	241408338~241409085
L3MBTL	chr20	+	NM_015478	41576211~41576660	41576111~41576660
LOC38945	chr7	+	NM_203393	5077964~5078727	5078079~5078727
				5078079~5078508	5078159~5078627
LOC38963	chr12	-	NM_00101298	8440516~8440670	8440516~8440670
LRP3	chr19	+	NM_002333	38377053~38377197	38376738~38377489
				38377053~38377489	38377053~38377694
LRRFIP1	chr2	+	NM_004735	238264797~238265347	238264597~238265449
MGC26718	chr18	+	NM_00102999	14168300~14169069	14168300~14169645
				14168300~14169249	14168300~14169755
MGC50273	chr2	-	NM_214461	132275706~132276378	
				132275906~132276280	132276121~132276476
MPP3	chr17	-	NM_001932	39265957~39266286	39265756~39266201
MPST	chr22	+	NM_021126	35744678~35745517	35745288~35745517
MRPL23	chr11	+	NM_021134	1924215~1924742	1924415~1924657
				1924300~1924742	
NCOR2	chr12	-	AK127788	123386915~123387562	123387128~123387562
NOXA1	chr9	-	NM_017820	139437582~139438226	139437667~139437931
					139436867~139437631
NQO2	chr6	+	NM_000904	2944533~2944882	2944638~2944882
NRG1	chr8	+	NM_013962	31616734~31617058	31616734~31617058
					31616624~31617268
NT5DC3	chr12	-	NM_016575	102758907~102759156	102758907~102759156
OXT	chr20	+	NM_000915	3000072~3000336	3000182~3000816
PELI3	chr11	+	NM_145065	65990881~65991345	65991076~65991245
PIWIL1	chr12	+	NM_004764	129387879~129388637	129388268~129388522
PNMT	chr17	+	NM_002686	35077644~35077868	35077644~35077868
				35077734~35077868	35077644~35078168
PODN	chr1	+	NM_153703	53300476~53300845	53300273~53300640
PODXL2	chr3	+	NM_015720	128830674~128831013	128830674~128831013
POMT2	chr14	+	NM_013382	76856814~76857059	76856609~76857269
PRG2	chr19	-	NM_024888	772845~773095	772845~773500
					772845~773095
PUS1	chr12	+	BC009505	130979949~130980613	130979844~130979998
RCOR2	chr11	-	NM_173587	63440933~63441690	63441043~63441292
					63441043~63441187
RIMS4	chr20	-	NM_182970	42872431~42872880	42872146~42872480
ROM1	chr11	-	NM_000327	62136237~62136983	62136237~62136983

Table S2. Selected 192 methylated genes in SCC and AC

Gene name	Chr	Strand	Refseq	Methylated region in AC	Methylated region in SCC
RPS21	chr20	+	NM_001024	60395433~60395768	60394918~60395282<br>60395433~60395768
SDF4	chr1	+	NM_016176	1156925~1157265	1156925~1157265
SENP6	chr6	+	NM_015571	76368652~76368906	76368062~76368511
SLC16A3	chr17	+	NM_004207	77779798~77780252<br>77779903~77780252	77779903~77780252
SORCS2	chr4	+	NM_020777	7245084~7245443	7244974~7245338
SOX8	chr16	+	NM_014587	971101~971445	970487~971445
SSTR4	chr20	+	NM_001052	22964041~22964290	22963821~22964375
TAF4	chr20	-	NM_003185	60074382~60074611	60074562~60074822
TBL1X	chrX	+	NM_005647	9392748~9392989	9392748~9392989
TCEA2	chr20	+	NM_198723	62158106~62158440	62158106~62158335
TCF7L1	chr2	+	NM_031283	85213755~85213999<br>85213950~85214214	85213855~85214114
TEX28	chrX	-	NM_001586	153176932~153177581<br>153177337~153177581	 153176932~153177216
TITF1	chr14	-	NM_003317	36059117~36059362	36059117~36059682
TKTL1	chrX	-	NM_012253	153176932~153177581<br>153177337~153177581	 153176932~153177216
TMEM151B	chr6	+	NM_00103970	44346056~44346890	44346146~44346399
TMEM178	chr2	+	NM_152390	39745797~39746346	39745797~39746261<br>39745997~39746659
TMEM32	chrX	-	NM_173470	134884030~134884604<br>134884134~134884604	 134883850~134884389
TNFRSF13C	chr22	-	NM_052945	40652503~40653256	40652503~40652662<br>40652413~40652662
TRIM17	chr1	-	NM_016102	226670954~226671384<br>226670954~226671599	226670653~226671188<br>226670653~226671278
TRIOBP	chr22	+	NM_007032	36472007~36472436	36472187~36472436
TYRO3	chr15	+	NM_006293	39638132~39638686<br>39638132~39638861	39638027~39638962
WDR26	chr1	-	NM_025160	222688910~222689159<br>222689030~222689159	 222688124~222689159
XPO4	chr13	-	AB051508	20374828~20375277	20374743~20375187<br>20374743~20375187
ZBTB7A	chr19	-	BC084568<br>NM_015898	 4018395~4018947	4016404~4016638<br>4017991~4018841
ZNF582	chr19	-	NM_144690	61596953~61597511<br>61597158~61597511	61596953~61597511<br>61597252~61597404
ZYX	chr7	+	NM_003461	142788593~142788848	142788593~142788753

AC, adenocarcinoma; SCC, squamous cervical carcinoma; Chr, chromosome; Strand, the direction of transcrition; +, the mRNA is transcripted from 5' to 3' direction; -, the mRNA is transcripted from 3' to 5' direction; Refseq, the NCBI RefSeq accession.
