# Supplementary material for: Methylomic Analysis Identifies Frequent DNA Methylation of Zinc Finger Protein 582 (ZNF582) in Cervical Neoplasms
Source: PLoS One. 2012 Jul 16;7(7):e41060. doi: 10.1371/journal.pone.0041060 (PMC3397950; doi:10.1371/journal.pone.0041060)
Supplement: Table S3 — The summary of polymerase chain reaction primers. (PDF) [file pone.0041060.s005.pdf]

Table S3. The summary of polymerase chain reaction primers

Primer Name	Forward Primer Sequence (5' - 3')	Reward Primer Sequence (5' - 3')	PCR SIZE (bp)	Annealing Temperature (℃)
QRT-PCR				
ADARB1_QR01	GCGATCCTTCCACCTCAACCTTCC	TTTTGGTCCGTAGCTGTCCTCTTG	96	60
ANKRD17_QR01	CAGTAGCAAGTCCTAAGCGTG	CCAGTAAACTCCCGAATAGCATT	151	60
B3GACT6_QR01	GCGCTTCGACACCGAATAC	CGTACACGTAGGACAGGCG	158	60
BAI2_QR01	ACAACCGAGATGAGATATGGTGA	GTCAGGGAACACACGCTCC	152	60
CASP9_QR01	GCGACCTGACTGCCAAGAAA	TCACAATCTTCTCGACCGACA	180	60
CRYGD_QR01	CCAGCCAGCCATGGGGAAGAT	CCGAGTTGCAGCGGCTCAAGT	116	60
CSPG5_QR01	GAGCTGACTTACCCATTTCAGG	CAGGGTGGTTCTCTGAGGTTC	151	60
DBC1_QR01	GTGTACGCTGTCGCCACAA	TCCCCAAAACCCATTCTCATTAC	113	60
ESRRA_QR01	CACTATGGTGTGGCATCCTGT	CGTCTCCGCTTGGTGATCTC	116	60
FOXF2_QR01	CGCCTCGCCTTACCTCAAG	CGTTCTGGTGCAAGTAGCTC	122	60
GAPDH_QR01	ACCCACTCCTCCACCTTTGACG	TCTCTTCCTCTTGTGCTCTTG	185	60
GNAQ_QR01	TGGGTCAGGATACTCTGATGAAG	TGTGCATGAGCCTTATTGTGC	144	60
GSTZ1_QR01	ATTTCTGACCTCATCGCTGGT	CTCCAGGGCGTTAAAGCCA	126	60
HPCA_QR01	GCGATGGCACCATAGACTTTC	GTCCATTTGGCGGAAGATTTTC	245	60
KIF1A_QR01	CCGGGAAATGAGCCGTGAC	GGTGTGCGACCAGTAGGAG	130	60
L3MBTL_QR02	GATTCCGCAGGAAGATTTCCA	TTGCACCTTACCTCGTCTTTG	255	60
LINGO1_QR01	GAACCGCATCAAAACGCTCAA	AAGACGCCTAGCGGGATGA	177	60
LRP3_QR01	ATCTTTCTCACCGGGAGACTC	GGGCTGTAGATGACCCCAC	101	60
MPP3_QR01	GGCGTCGCTGTTCCACATA	CCAGGCTGGTTCCATACAGAT	160	60
MPST_QR01	TTCGACATCGACCAGTGCAG	CGTACTCCGCGAAATGCTC	79	62

Table S3. The summary of polymerase chain reaction primers

Primer Name	Forward Primer Sequence (5' – 3')	Reward Primer Sequence (5' – 3')	PCR SIZE (bp)	Annealing Temperature (℃)
MRPL23_QR01	CCCACAACTTCGGGTGTTCC	CCACGGGCACGTTATAGATG	155	60
MSI1_QR01	GGACTCAGTTGGCAGACTACG	CTGGTCCATGAAAGTGACGAA	138	60
NKX2-1_QR01	CTCAGCCGCCGCCGAATCA	CCGCCCTCCATGCCCACTT	113	60
NT5DC3_QR01	CAGACCTTGACTGGCTTATTGG	GGTCATTTCTCGCATCTCCTTC	108	60
PDE8B_QR01	AACTCGCTGATCTGCCCAAAA	CTGGCATAGTAAACCCCCTGC	106	62
PELI3_QR01	ACCAATGGAGTCCTGGTGATG	AGGTCGATGAGAGAGCCGTC	185	60
POMT2_QR01	TTCTGTGCATTCCTTGGCTCC	AGAGTGAGGCATCCCGTGT	128	60
RBM35B_QR01	CGACTTAATCCTCCTAGTTTGGC	GCACGAACCAGCGATTTGT	81	60
SDF4_QR01	GAGGCCATGGAGGAGAGCAAGACACA	AACCTCCTTCTCGCTATGGCCTTTAC	123	60
SRD5A2_QR01	CACAGGACATTTGTGTACTCACT	GTGTACCACCCATCAGGGTAT	149	60
SYNGR2_QR01	GACGCGTATTTCCCCCAGATCAGC	TCACAGAGTCGGCCCCCACCAG	172	60
TCF7L1_QR01	ACTATTTCGCCGAAGTGAGAAG	GGGGTCCGTTGGAGAGGTA	120	60
TST_QR01	TCCCTGCTCAAGACCTACGAG	GCCCGAGTCCAGTCCTACT	132	60
WDR26_QR01	CCTGGAGGCACTTCAAGTTCT	GCTTTTGCACGTAGGTCTTCT	120	60
ZNF582_QR01	ATGTCCCTTGGGTCAGAATTGT	TTGCCTTGCTCTAGGAAGGAG	185	60
ZYX_QR01	TCTCCCGCGATCTCCGTTT	CCGGAAGGGATTCACTTTGGG	102	60
Methylation Specific PCR (MSP)				
ADARB1_MS04	ACGAAGGCGGGTAGAGTTTTTTAGTC	CTCCCTCACGACCACCTACGAC	174	61
ANKRD17_MS01	GCGTTCGCGGGGTTTTAGATTC	CTCTTTCCTCGCTATTTCCCGCA	188	62
B3GALT6_MS02	TCGGTTTGTTTTTGTTTTCGGGAATTTC	CCTCCCGCCTTAAACTAAAACATCGTC	106	59
CASP9_MS01	GGTTTTGGAGATGCGTTCGGAGGC	CTTCCACCAACCGCAACCGAC	216	60

Table S3. The summary of polymerase chain reaction primers

Primer Name	Forward Primer Sequence (5' - 3')	Reward Primer Sequence (5' - 3')	PCR SIZE (bp)	Annealing Temperature (℃)
CBFA2T3_MS03	GAAAGTTCGGTTGATTGAGGGTTAGC	CACCCACCCTAAACCCTACAACG	210	58
CBFA2T3_MS06	TCGTGAGTCGGTTTTGGGTTTGC	CTTCCTAAAAAAAAACAATAACCCTTCG	134	60
COL2A1	GGGAAGATGGGATAGAAGGGAATAT	AACAATTATAAACTCCAACCACCAAAC	91	60
CRYGD_MS02	GTTTTTTTTCGGGGGTTTTTTTTGTGC	TAAAAAAAACTCCGAAATCCCGAAACG	187	60
CSPG5_MS03	GTTATGGCGGGTTTGGTTGTGC	CAAAACCTAACAAAAAAACGAAACCGA	113	60
ESRRA_MS01	GTTGCGGGGCGGGATTTGTAC	AAAATAAAACCGACCGTACGCAT	158	61
GNAQ_MS01	GGTGGGCGCGGATAGGGTC	TCACCGAAATATCCCCGCAACGA	116	61
GNB1_MS01	GGTTTTTCGGGGTAGCGGTATTC	CGAAAAACCCCACGCGCTC	175	61
KIF1A_MS02	GGTTTAGAGCGGGGGTAGGTGGAC	CGAAAAATAAAAAACCGACACCCGA	209	58
KIF1A_MS06	TGGGACGGTGGAGGGAGGAC	CAAATCACACGCCGCTCGAA	129	58
L3MBTL_MS03	TTTTGTCGTAGTTTAGCGTCGGGTC	AACCCCGCCCCATAACTACGA	160	62
LINGO1_MS02	GGATTGTCGGGCGGAGGTGTC	ACCGCCTAACTCACCTACATCTCGAA	152	60
LRP3_MS04	GGTTGGTGCGTAGAGTTGTTTAGTATTTC	CCGAATACCGCCGATAAAACGTC	207	58
MPP3_MS04	GGGATTTAGGGAGCGGGAGAGC	TCCCGAACCCCTCCCTACGAC	202	64
MPST_MS03	GTTAGAGGGGGATTTTGCGTCGTTC	CCCCTCAAACCCCCGCGAAT	120	64
NKX2-1_MS02	ATTCGGGATAGTTTTCGGGAGGTAGTC	GTCAAAAAAACACCTCTACTCCCCGC	112	60
NKX2-1_MS13	GGGTGTTGGGGTTGTGATGTTTTC	AACAAACCTAATATCCCTAACCCTCGC	96	58
NT5DC3_MS09	TAGTTTTAGAAGGTAGGACGAAGGTGC	CCCACGCCCAACCTACTACGA	153	61
PDE8B_MS01	GAGGTTCGGCGGGGGGTATC	CCGCTACCACCTACAATAAAAAAACGAC	112	61
PELI3_MS04	AGGGAGTTTTTTAGACGGAGTGGAGAGC	CCAAACTCCGCCTCTTACGACGAT	136	60
POMT2_MS06	GGAATTTCGAGTTTTAGCGGGTAGC	CAAACCGACAACGACTCCCGACAT	92	68

Table S3. The summary of polymerase chain reaction primers

Primer Name	Forward Primer Sequence (5' – 3')	Reward Primer Sequence (5' – 3')	PCR SIZE (bp)	Annealing Temperature (℃)
RBM35B_MS01	GTTTTGGCGGTTTTTTTTTAAGGTTTTC	ACCCACGCCCTCTCCAAACG	91	60
SDF4_MS01	AGGTTTTCGTGTCGCGTTTGTTTTC	CGCAACGTAATCCGCAACACGTA	140	68
SSTR4_MS06	GGGATTTCGGGAGGGTCGGAC	CACCCCAACTCCTAAAAATCAACGC	178	60
SYNGR2_MS03	GATTTTTTTTGGTTCGTGTTGCGGTC	GACTCCACCCGCCGAAAACGTA	203	59
TCF7L1_MS04	TTTCGTAGGGGGTAGTGTCGTTCGTTC	ACGCCCACCCCTCCGCAT	117	66
WDR26_MS08	TATGTACGTTCGGTTTCGGAGGGC	CCCATCCACGACCCCGACT	129	60
ZYX_MS03	GTCGGAGGCGTATAGGGGTTGC	CGAATACCCGAAATCTAACTCACCGTC	101	61
Unmethylation Specific PCR (U-MSP)				
PDE8B_UM04	TGTGTGTTTTTTTGTTTGGGGAGGAAGA	CCAACCTATATATCCACCACTCCATC	126	60
Bisulfited Sequencing PCR (BS-PCR)				
PDE8B_BS01	GAAAGTGGGGAAAGAAGGTGTAGGT	CTAAAAACRCAACCCATCCCTC	464	60
