# Supplementary material for: Methylomic Analysis Identifies Frequent DNA Methylation of Zinc Finger Protein 582 (ZNF582) in Cervical Neoplasms
Source: PLoS One. 2012 Jul 16;7(7):e41060. doi: 10.1371/journal.pone.0041060 (PMC3397950; doi:10.1371/journal.pone.0041060)
Supplement: Material and Methods S1 — (DOC) [file pone.0041060.s006.doc]

# Supplementary Material and Methods

## Title

Methylomic Analysis Identifies Frequent DNA Methylation of *Zinc Finger Protein 582* *(ZNF582)* in Cervical Neoplasms

## Authors and Affiliations

Rui-Lan Huang^1,2,3☯^, Cheng-Chang Chang^1,3,4☯^, Po-Hsuan Su^1,4^, Yu-Chih Chen^1,4^, Yu-Ping Liao^1,5^, Hui-Chen Wang^1,3^, Yi-Te Yo^1,3^, Tai-Kuang Chao^6^, Hsuan-Cheng Huang^2^, [Ching-Yu Lin](http://mts.tmu.edu.tw/people/bio.php?PID=15)^7^, Tang-Yuan Chu^8^, Hung-Cheng Lai ^1,3,4,5*^

^1^Department of Obstetrics and Gynecology, Tri-Service General Hospital, Taipei, Taiwan,

^2^Institute of Biomedical Informatics, National Yang-Ming University, Taipei, Taiwan

^3^ Laboratory of Epigenetics and Cancer Stem Cells, National Defense Medical Centre, Taipei, Taiwan

^4^Graduate Institute of Medical Sciences, National Defense Medical Center, Taipei, Taiwan,

^5^Graduate Institute of Life Sciences, National Defense Medical Center, Taipei, Taiwan,

^6^Department of Pathology, Tri-Service General Hospital, Taipei, Taiwan

^7^School of Medical Laboratory Science and Biotechnology, College of Medical Science and Technology, Taipei Medical University, Taipei, Taiwan

^8^Center for Cervical Cancer Prevention, Department of Obstetrics and Gynecology, Graduate Institute of Clinical Medicine, Tzu Chi Medical Center, Tzu Chi University, Hualien, Taiwan

## Correspondence to:

Hung-Cheng Lai, M.D., Ph.D.,

Department of Obstetrics and Gynecology, Tri-Service General Hospital, National Defense Medical Center, 5F, 325, Sec 2, Cheng-Gong Rd., Neihu district, Taipei city 114, Taiwan

TEL: +886-2-8792-7199.

Fax: +886-2-8792-7199.

*E-mail: hclai@ndmctsgh.edu.tw

☯These authors contributed equally to this work.

Material and methods

MeDIP-on-chip

Genomic DNA (10 μg) in 90 μL of nuclease-free water was fragmented by sonication to sizes of about 300–500 base pairs (bp).The MeDIP assay was performed with 4 μg of the fragmented genomic DNA in immunoprecipitation buffer (0.15% SDS, sodium phosphate [pH 7.0], 150 mM NaCl, 1 mM EDTA [pH 8.0], 0.5 mM EGTA [pH 8.0], 10 mM Tris-HCl [pH 8.0], 0.1% bovine serum albumin, and 0.1% Triton X-100). Anti-5-methylcytosine antibody (30 μg; Abcam, ab1884, MA, USA) and 7 mM NaOH were added and incubated overnight in 100 μL of immunoprecipitation buffer, after which the DNA/antibody complexes were collected with 120 μL of Protein G Sepharose beads (Amersham GE, PA, USA) for 2 h. The beads were washed twice with low-salt buffer (0.1% SDS, 1% Triton X-100, 2 mM EDTA, 20 mM Tris-HCl [pH 8.1], 150 mM NaCl), once with high-salt buffer (0.1% SDS, 1% Triton X-100, 4 mM EDTA, 20 mM Tris-HCl [pH 8.1], 150 mM NaCl), once with lithium chloride buffer (0.25 M LiCl, 0.5% NP-40, 0.5% deoxycholate, 1 mM EDTA, 0.5 mM EGTA, 10 mM Tris-HCl [pH 8.0]), and twice with 1 mL of TE buffer (10 mM Tris-HCl, 1 mM EDTA [pH 8.0]) at 4 °C. The immunoprecipitation-enriched DNA was eluted with freshly prepared 1% SDS and 0.1 M NaHCO_3_, then purified with phenol/chloroform extraction, ethanol precipitated, and redissolved in 40 μL of elution buffer (10 mM Tris-HCl [pH 8.5]). The input samples were collected from the supernatant after immunoglobulin G (IgG) immunoprecipitation and processed in parallel. All chemicals were made by Sigma-Aldrich. The enriched DNA and input DNA were amplified with the Whole Genome Amplification Kit (Sigma), according to the manufacturer’s instructions, to generate the libraries.

Public microarray integration

Gene expression data, GSE7803, was operated using clinical samples and had been published by Zhai Y *et al.* . Raw data were processed using the quintile normalization and fold change analyses. Gene expression greater than 1.2 folds in normal tissues relative to cancer was selected for further analysis. The tissue differential methylation regions (T-DMRs) from the supplemantary data, located at promoter regions were included for analysis (<http://www.nature.com/ng/journal/v41/ n2/suppinfo/ng.298_S1.html>) .

Methylation-specific PCR (MSP) and bisulfite sequencing

The MSP primers and annealing temperatures are listed in Table S3. MSP was performed in a total volume of 11 μL, containing 1 μL of bisulfite-converted DNA, 150 nM each primer, and 1 × Gold PCR Master Mix (Bionovas, Toronto, CA). MSP amplification was performed under the following cycling conditions: denaturation at 95 °C for 10 min, 38 polymerization cycles (95 °C for 30 s, annealing at the appropriate temperature for 30 s, and 72 °C for 30 s), and final extension at 72 °C for 5 min. The PCR conditions for bisulfite sequencing were the same as for MSP, and amplified in a total volume of 36 μL. We used the PCR Advanced Kit (Viogene, Taiwan) and the T&A Cloning Vector Kit (RBC Bioscience, Taiwan) to purify and clone the PCR products, respectively. At least five individual clones for each sample were subjected to bisulfite sequencing.

### Supplementary Figure legends

Figure S1. The intensity of probes by MeDIP-on-chip for thirty-two regions (including 35 genes) in cervical tissue.

The arrows indicated the direction of mRNA transcription. One bar represents one probe. The Y-axis shows the value of the –log10 *P-*value by the KS test for each probe. SCC, squamous cervical carcinoma; AC, adenocarcinoma and N, normal cervical scraping cells. The light-blue spotlight lines show the transcription star sites. Almost all probes around these promoters presented high signals in tumor tissues.

Figure S2: Genes re-expression analysis by QRT-PCR in cervical cell lines

The Y-axis represents fold changes of gene expression analysis after demethylation treatment of cervical cancer cell lines. DAZ, 5-aza-2′-deoxycytidine; TSA, trichostatin A.

Supplementary Table

Table S1. Demographic for discovery and pre-validation phase studies

Table S2. Selected 192 methylated genes in SCC and AC

Table S3. The summary of polymerase chain reaction primers

## Reference:

1. Zhai Y, Kuick R, Nan B, Ota I, Weiss SJ, et al. (2007) Gene expression analysis of preinvasive and invasive cervical squamous cell carcinomas identifies HOXC10 as a key mediator of invasion. Cancer Res 67: 10163-10172.

2. Irizarry RA, Ladd-Acosta C, Wen B, Wu Z, Montano C, et al. (2009) The human colon cancer methylome shows similar hypo- and hypermethylation at conserved tissue-specific CpG island shores. Nat Genet 41: 178-186.
